# Effects of intermittent fasting combined with exercise on serum leptin and adiponectin in adults with or without obesity: a systematic review and meta-analysis of randomized clinical trials

**DOI:** 10.3389/fnut.2024.1362731

**Published:** 2024-06-12

**Authors:** Fatemeh Kazeminasab, Nasim Behzadnejad, Henrique S. Cerqueira, Heitor O. Santos, Sara K. Rosenkranz

**Affiliations:** ^1^Department of Physical Education and Sport Sciences, Faculty of Humanities, University of Kashan, Kashan, Iran; ^2^Department of Exercise Physiology, Faculty of Physical Education and Sport Sciences, University of Isfahan, Isfahan, Iran; ^3^School of Medicine, University of São Paulo, Ribeirão Preto, Brazil; ^4^Postgraduate Program, Faculdade UNIGUAÇU, Cascavel, Brazil; ^5^Department of Kinesiology and Nutrition Sciences, University of Nevada Las Vegas, Las Vegas, NV, United States

**Keywords:** adiponectin, adipokine, exercise training, intermittent fasting, leptin

## Abstract

**Context:**

Intermittent fasting (IF) and exercise training (Exe) have been evaluated in several studies for improving cardiometabolic biomarkers related to weight loss. However, further investigation is required to understand the potential effects on leptin and adiponectin concentrations. IF protocols have been shown to be efficient in improving adipokines, but further research is required to determine whether or not IF regimens combined with Exe are superior to Exe alone.

**Objective:**

The aim of this study was to determine whether or not interventions combining IF plus Exe are more effective than Exe only for improving serum leptin and adiponectin in adults with and without obesity.

**Data extraction:**

A systematic review and meta-analysis was performed by searching PubMed, Scopus, and Web of Science databases up to August 2023 for randomized clinical trials that determined the effects of IF plus Exe vs. Exe alone (control) on body weight, serum leptin, and serum adiponectin. Analyses were conducted for IF plus Exe vs. Exe alone to calculate weighted mean differences (WMD) and standardized mean differences (SMD).

**Analysis:**

The current meta-analysis included 6 studies with a total sample of 153 participants, with intervention durations ranging from three days to 52 weeks. IF plus Exe elicited significantly larger decreases in leptin levels [SMD = −0.47, *p* = 0.03], which were accompanied by weight loss [WMD = −1.25 kg, *p* = 0.05], as compared with exercise-only interventions, but adiponectin did not differ between the two [SMD = 0.02, *p* = 0.9].

**Conclusion:**

IF combined with Exe reduced leptin significantly, but did not change adiponectin levels, when compared to exercise only. Perhaps these reductions in leptin levels may have been associated with weight loss; however, due to the small number of included studies and the high heterogeneity in the weight loss outcomes, this result is uncertain.

**Systematic review registration:**

https://www.crd.york.ac.uk/prospero/, identifier CRD42023460735.

## 1 Introduction

In this century, two billion people in the world are facing obesity (1), which is a low-grade inflammatory disease associated with cardiovascular diseases, diabetes, cancer, and osteoarthritis (2, 3). The inflammation inherent to obesity is caused by white adipose tissue upon prolonged activity of the innate immune system (4). Adipose tissue acts like an endocrine gland by producing biologically active hormones called adipokines, which include leptin and adiponectin (5).

Leptin is well-established as a hormone that increases satiety and energy expenditure. Serum leptin concentrations are associated with variable hunger or satiety states (6). In the brain, leptin signals exclusively through its long isoform receptor (LepRl) and participates in several neuroendocrine systems. Experimental research has reported a selective reduction of LepRls expressed in specific subgroups of neurons in certain hypothalamic and extrahypothalamic regions of the central nervous system due to high circulating leptin concentrations (7). Clinically, in people with obesity, increased leptin levels are associated with leptin resistance, resulting in a decrease in the appetite-suppressing effects of leptin. Some evidence suggests that central leptin resistance causes obesity and that obesity-induced leptin resistance may cause injury to peripheral tissues (7). In addition, the loss of leptin-LepRl signaling in the brain is sufficient to promote obesity (8).

Changes in leptin and adiponectin should be considered together. Interestingly, weight loss may result in improvements in circulating leptin/adiponectin levels and in leptin/adiponectin sensitivity, ultimately enhancing appetite control (8). Obesity leads to a decrease in adiponectin levels, a phenomenon associated with insulin resistance (9). Plasma leptin concentration is also directly related to obesity, such that the amount of leptin increases with increases in fat tissue, leading to obesity-associated hyperleptinemia (10). Such dysregulation favors the cardiovascular and metabolic disorders in people with obesity. More specifically, adiponectin exerts an indirect action in insulin-sensitizing via receptors R1/R2 to stimulate signaling pathways (mainly AMPK); elicits anti-inflammatory effects through modulating PPARα, MAPK, and NF-kB; and plays an antiangiogenic effect by hindering basic fibroblast growth factor and interleukin-8 (11). Leptin, in turn, is an endocrine hormone derived from fat cells with paradoxical effects (12); despite its fundamental protective role in the cardiovascular system, there are detrimental mechanisms in obesity models whereby leptin is associated with elevated oxidative stress and decreased nitric oxide bioavailability in endothelial cells, facilitating thrombosis formation and atherosclerosis via hypertrophy, proliferation, migration, and calcification of vascular smooth muscle cells (13).

The reported effects of intermittent fasting (IF) and calorie-restricted diets on the levels of these two adipokines are contradictory (14). These diets may lead to decreases in leptin mRNA and protein levels, while having no effect on adiponectin mRNA levels (14, 15). However, some studies have reported increased leptin levels and/or no changes (16). Furthermore, other studies have reported increased adiponectin levels following IF (15), or decreased adiponectin levels during one month of Ramadan fasting (17).

Reductions in inflammatory factors during fasting, result in increases in lipoprotein lipase and fatty acid use. Due to limited access to glucose, fatty acids are used as a substitute fuel source, leading to fat mass loss in conditions of negative energy balance. The release of adiponectin is associated with decreases in concentrations of fatty acids and triglycerides in circulation, subsequent to increased fatty acid oxidation (6). Stimulation of intracellular lipolysis during fasting is triggered by changes in the plasma levels of several hormones, including decreased insulin levels and increased catecholamines, cortisol, and growth hormone. Additionally, sympathetic nerve innervation of adipose tissue stimulates lipolysis in a pulsatile manner during fasting. Reductions in leptin levels, as a result of prolonged fasting, primarily affect the neuroendocrine system and may have limited direct effects on metabolism (16).

There are different types of IF regimens that should be considered, for example, alternate day fasting (ADF) and time-restricted feeding (TRF), where ADF alternates between feeding and fasting days, and TRF includes a daily fasting window of 12–22 h (18, 19). IF diets have emerged in the management of cardiometabolic disorders in rodents and humans (1, 5, 20–24). In addition, the feeding time restriction appears to be a dietary strategy related to the modulation of circadian rhythm and energy metabolism of different organs by orchestrating the secretion of insulin, adiponectin, and leptin while reducing body fat (25–27). Not surprisingly, IF regimens have gained attention in the fields of medicine and sports, in which IF combined with exercise (Exe) has been shown to be a useful non-pharmacological tool to improve body composition (fat loss and muscle maintenance) and markers of cardiovascular and metabolic health (5, 18, 28).

Previous research indicates that acute exercise alone (< 12 weeks) is not effective for reducing leptin levels in obesity, with greater benefits following moderate- and high-intensity aerobic exercise as well as resistance training performed for > 12 weeks (3–4 times a week) (29). In a meta-analysis by Fedewa et al. (30), reductions in leptin levels were observed with at least two weeks of Exe (30). Regarding adiponectin, aerobic exercise along with resistance training can increase adiponectin levels in people with type 2 diabetes or metabolic syndrome (31, 32).

Nevertheless, the effects of IF diets combined with Exe must be further investigated with respect to leptin and adiponectin levels, as these markers are important to elucidating cardio-metabolic outcomes. The present systematic review and meta-analysis was conducted to examine the effects of combining IF diets with Exe as compared with Exe alone, on circulating leptin and adiponectin levels in humans with or without obesity and obesity-related diseases.

## 1.1 Trial registration

The current systematic review and meta-analysis was registered in the PROSPERO International Prospective Register of Systematic Reviews, ID: CRD42023460735, and was conducted according to The Preferred Reporting Items for Systematic Reviews and Meta-analysis (PRISMA) guidelines (33) and guidance provided in the Cochrane Handbook of Systematic Reviews of Interventions (34).

## 1.2 Search strategy

Original articles (research articles) published from inception through August 2023 were identified using electronic database searches, including Scopus, PubMed, and Web of Science. Two reviewers independently recognized published research papers. The keywords “Intermittent energy restriction” or “intermittent caloric restriction” or “intermittent fasting” or “fasting” or “intermittent energy” or “intermittent calorie” or “intermittent diet” or “time-restricted feeding” or “Time Restricted eating” or “alternate-day fasting” or “alternate day fasting” or “alternate day diet” or “Ramadan fasting” were utilized for conducting the searches.

Furthermore, the search strategy aimed at identifying studies that incorporated exercise training encompassed keywords such as “Exercise” or “training” or “physical activity” or “exercise training” or “sport” or “strength training” or “strength exercise” or “weight training” or “resistance training” or “progressive training” or “progressive resistance” or “weightlifting” or “aerobic exercise” or “aerobic training” or “endurance exercise” or “endurance training” or “cardio training” or “physical endurance” or “physical exertion,” and for adipocytokines, keywords included a combination of “adipokine” or “adipocytokine” or “leptin” or “adiponectin.”

To ensure comprehensive coverage of relevant records, the reference lists of all included studies were examined for any additional sources that may have been missed in the initial electronic searches. The searches were limited to articles written in English and randomized control trial (RCT) studies involving adult human participants aged between 18 and 65 years. There was no limit on publication dates. In addition, further searches were accomplished in Google Scholar as well as hand searches of reference lists from all retrieved studies for additional articles. Supplementary Table 1 shows the search strategies utilized for the included databases.

## 1.3 Study selection and inclusion criteria

**FIGURE 1:**
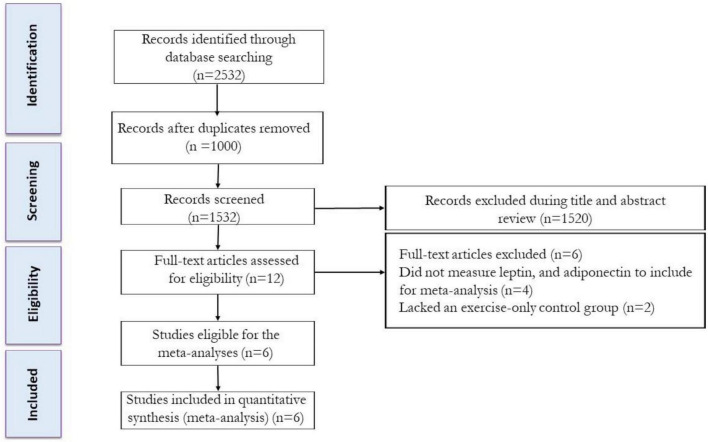
Flow diagram of systematic literature search.

Studies were included if they met the following criteria: (a) peer-reviewed, full-text articles, and (b) trials that included the impacts of IF plus Exe vs. Exe alone (control) on serum leptin and adiponectin in adults with or without overweight and obesity. The process of selecting the studies is shown in Figure 1. After eliminating duplicate studies, the titles and abstracts of articles were evaluated separately. Next, two reviewers independently examined the complete texts of potentially suitable studies to assess their eligibility. Any disagreements were resolved by discussing with another author. The study characteristics that were extracted included: (A) participant characteristics including age, biological sex, body mass index (BMI), health status, and sample size; (B) study design; and (C) IF and Exe characteristics, and intervention duration (weeks).

Two authors extracted these data. Forest plots were generated by conducting meta-analyses using the pre- and post-intervention means and standard deviations, or mean differences and their corresponding standard deviations for each outcome (such as body weight, serum leptin, and adiponectin). In cases where the means and standard deviations (SDs) were not provided, the SDs were calculated from the standard errors of means (SEM), medians and interquartile ranges (IQRs), or means and IQRs (35–37).

## 1.4 Quality assessment and sensitivity analyses

The assessment of potential bias was conducted using the PEDro [Physiotherapy Evidence Database tool (38)]. We excluded two items (for no blinding of participants and no blinding of intervention providers) from the original 11-item scale because participants and intervention providers could not be blinded to the assigned diet and Exe conditions during studies. The current study employed a scale comprising nine elements: (1) defined inclusion and exclusion criteria for eligibility, (2) randomized participant allocation, (3) concealed allocation, (4) similarity of groups at baseline, (5) blinding of all assessors, (6) evaluated outcomes in 85% of participants, (7) intention-to-treat (ITT) analysis, (8) reporting of statistical comparisons between groups, (9) and point measures and measures of variability (Supplementary Table 2).

Using sensitivity analyses by removing one study at a time, the dependence of the findings on individual studies was determined and the impact of each study on the overall results of the meta-analysis was assessed.

## 1.5 Statistical analysis

The Comprehensive Meta-analysis version 2.0 (software developed by Biostat Inc., New Jersey, USA) was used to conduct meta-analyses. These analyses involved calculating weighted mean differences (WMD), standardized mean difference (SMD), along with 95% confidence intervals (CIs), to assess outcomes. The calculations were performed using random effects models. Effect sizes were calculated to evaluate and compare the impacts of combining IF with Exe vs. Exe alone on adipokines including body weight, serum leptin, and adiponectin. Interpretation of effect sizes was conducted: 0.2–0.49 indicates small effects, 0.5–0.79 indicates moderate effects, and > 0.80 indicates large effects. To assess heterogeneity, the *I*^2^ statistic was quantified, with a significance level set at *p* < 0.05. As per the guidelines provided by Cochrane, the interpretation of *I*^2^ statistics was defined as follows: 25% indicates low heterogeneity, 25–50% suggests moderate heterogeneity, 50–75% signifies high heterogeneity, and ≥ 75% indicates considerable heterogeneity (39). The findings were combined using random effects models, taking into consideration the possibility of heterogeneity in clinical or methodological factors that could have influenced the outcomes (40).

Publication bias was assessed using visual interpretation of funnel plots. In cases where publication bias was found, Egger’s tests were employed as a supplementary confirmatory measure. If the *p*-value was < 0.1, it was determined that there was considerable evidence of publication bias (39).

## 2 Results

### 2.1 Included studies

From our initial search strategy, we found a total of 670 records in PubMed, 978 records in Scopus, and 884 records in Web of Science. After excluding duplicate records and assessing the titles and abstracts, 12 studies were determined to be relevant and necessitated a comprehensive assessment of their complete texts. After conducting a detailed assessment of the full texts, six studies were excluded for the following reasons: (A) Did not measure body weight, leptin, or adiponectin (*n* = 4), and (B) Lacked a control group (exercise alone) (*n* = 2). In this systematic review and meta-analysis, a total of 6 studies were appraised, which comprised of 6 intervention groups involving the combination of IF and Exe. The flow diagram of the systematic literature search is shown in Figure 1.

### 2.2 Participant characteristics

A combined group of 153 adults with obesity or healthy trained participants were included, with sample sizes ranging from 10 (1) to 42 (5). The average age and BMI varied across the studies. The ages ranged from 22.5 years (18) to 65 years (5), while the BMIs ranged from 22.5 kg/m^2^ (1) to 37 kg/m^2^ (5). A total of six studies were included in the meta-analysis. Among them, five studies only included male participants (1, 18, 25, 28, 41), and one study had both male and female participants (5). Participant health status varied across the studies. Some studies included healthy participants (1, 18, 25, 28, 41) while one study focused on participants with obesity (5). For more detailed information on participant characteristics, please refer to Table 1.

**TABLE 1:** Study, participant, and intervention characteristics.

	Study characteristics				Participant characteristics						
Reference	Sample size (biological sex)	Groups	Intervention duration	Outcomes	Health status	Age (years) Mean ± SD	BMI (kg/m^2^) Mean ± SD	Intermittent fasting characteristics	Non-fasting control eating	Exercise characteristics	Energy intake (kcal/day)
Bhutani et al. (5)	42 M&F	ADF + Exe Exe alone	12 weeks	Leptin Adiponectin	Obesity	ADF + Exe: 25–65 Exe alone: 25–65	ADF + Exe: 30–39.9 Exe alone: 30–39.9	ADF: During weeks 1–4, participants consumed 25% of their baseline energy needs on the “fast day” (24 h) and consumed food *ad libitum* on each “feed day” (24 h) During weeks 5–12, subjects continued with the ADF regimen but no fast-day food was provided to them	Subjects were not given any dietary counseling and maintained their regular eating habits	Aerobic Exe: 25–40 min exercise training at 60–75% MHR 3 times/week	NM
Cherif et al. (41)	21 M	IF + Exe Exe alone	3 days	Leptin Adiponectin	Active males	IF + Exe: 29.8 ± 5.9 Exe alone: 29.8 ± 5.9	NM	IF: No food or drink from dawn (∼4:00 a.m.) to sunset (∼4:45 p.m.), fasting duration was approximately 14 h	Normal fed state (last meal 5 h before the experimental trial and no drink after arrival at the laboratory	Aerobic Exe: 2 sets with 5 × 5-s maximal sprints with 25 s of recovery between sprints and 3 min of recovery between sets on an instrumented treadmill	NM
Moro et al. (25)	20 M	TRE + Exe Exe alone	52 weeks	Leptin Adiponectin	Healthy males	NM	NM	TRF: 16-h fasting and 8-h feeding (16/8); All calories between 12:00 p.m. and 8:00 p.m.); consume the total caloric needs divided into three meals eaten in an 8-h window (∼1 PM, 4 PM, and 8 PM),	Normal diet	Resistance Exe: thrice-weekly training schedule, training intensity: 75–90% of 1RM	Exe: 2,978.0 ± 245.1 IF + Exe: 2,580.0 ± 245.3
Moro et al. (28)	34 M	TRF + Exe Exe alone	8 weeks	Leptin Adiponectin	Healthy, resistance-trained males	TRF + Exe: 29.94 ± 4.07 Exe alone: 28.47 ± 3.48	NM	TRF: 16-h fasting and 8-h feeding (16/8); three meals consumed at 1 p.m., 4 p.m., and 8 p.m. for each day. The distribution of calories was 40, 25, and 35% at 1 p.m., 4 p.m. and 8 p.m. respectively	Normal diet	Resistance Exe: 3 × of 6–8 repetitions at 85–90% 1RM, 3 times/week	Exe: 3,007 ± 444.7 IF + Exe: 2,826 ± 412.3
Stratton et al. (18)	26 M	TRF + Exe Exe alone	4 weeks	Leptin Adiponectin	Healthy, resistance-trained males	TRF + Exe: 22.9 ± 3.6 Exe alone: 22.5 ± 2.2	NM	TRF: All calorie and macronutrient consumption occurring within an 8 h period each day and a prescribed 25% caloric deficit	Normal diet	Resistance Exe: the 4-week training protocol consisted of full body sessions at 3–8 repetitions, 3 times/week	Exe: 1,939 ± 260 IF + Exe: 1,946 ± 310
Harder-Lauridsen et al. (1)	10 M	IF + Exe Exe alone	4 weeks	Leptin Adiponectin	Healthy lean men	25.2 ± 35.2	22.5 ± 31.36	Ramadan model IF: 12-h overnight fast	Normal diet	Aerobic Exe	Exe: 2,606 IF + Exe: 2,380

ADF, alternate day fasting; Exe, exercise; F, female; IF, intermittent fasting; M, male; NM, not mention; TRF: time-restricted feeding.

### 2.3 Intervention characteristics

A combination of ADF, TRF, IF, and Ramadan IF methodologies were included among the studies included in the analysis. One study used ADF with one day of feeding and one day of fasting (5); three studies employed TRF with a 16-h fasting period and an 8-h feeding window (18, 25, 28). Additionally, two studies followed IF which involved a 10–12-h eating window and a fasting period of 12–14 h on fasting days (1, 41). On those fasting days during weeks 1–4, participants consumed only 25% of their estimated energy intake (5). One study had a TRF regimen with a 25% caloric deficit during an 8-h feeding period (18). Two studies used a TRF regimen with three meals consumed at 1 p.m., 4 p.m., and 8 p.m. each day. The distribution of calories was 40, 25, and 35% at 1 p.m., 4 p.m. and 8 p.m., respectively (25, 28). These regimens were maintained for durations of 3 days (41), 4 weeks (1, 18), 8 weeks (28), 12 weeks (5), and 52 weeks (25). In all cases, the control groups refrained from IF and solely engaged in Exe. The studies incorporated various forms of Exe interventions, including aerobic Exe (1, 5, 41), and resistance training (18, 25, 28). One study included 25–40 min aerobic Exe at 60–75% maximum heart rate (5), one study used Exe based on 2 sets with 5 × 5-s maximal sprints with 25 s of recovery between sprints, and 3 min of recovery between sets on an instrumented treadmill (41). Three studies included resistance training at an intensity of 75–90% 1RM (18, 25, 28). One study did not report the intensity and duration of each Exe session (1). Detailed information about the intervention (IF and Exe) characteristics can be found in Table 1.

### 2.4 Meta-analysis

#### 2.4.1 The effects of IF plus Exe on body weight in adults

**FIGURE 2:**
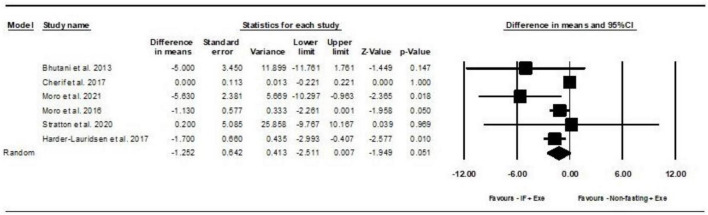
Forest plot of the effects of intermittent fasting plus exercise vs. exercise only on body weight. Data are reported as WMD (95% confidence limits). WMD, weighted mean differences; IF, intermittent fasting; Exe, exercise.

Based on 6 intervention arms, IF plus Exe decreased body weight [WMD = −1.25 kg (95% CI −2.51 to 0.007), *p* = 0.05] significantly more than Exe only (Figure 2). The studies included in the analysis demonstrated high heterogeneity (*I*^2^ = 71.05%, *p* = 0.004). The absence of publication bias was supported by the results of funnel plots and the Egger’s test (*p* = 0.5). The sensitivity analysis conducted by excluding individual studies demonstrated that for five of the studies, there were no alterations in the effect size, significance of the findings, or the direction of the results. By excluding the (41) study during sensitivity analysis, the effect size and significance of the results were altered (WMD = −1.65 kg, *p* = 0.002), but the overall findings remained consistent.

#### 2.4.2 The effects of intermittent fasting plus Exe on adiponectin in adults

##### 2.4.2.1 Leptin

**FIGURE 3:**
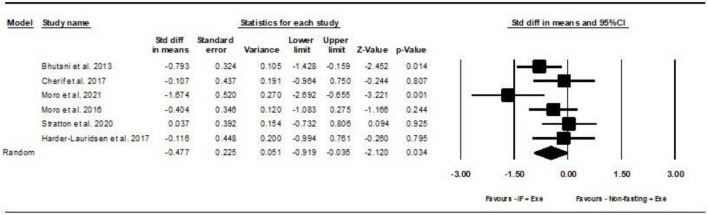
Forest plot of the effects of intermittent fasting plus exercise vs. exercise only on serum leptin. Data are reported as SMD (95% confidence limits). SMD, standardized mean difference; IF, intermittent fasting; Exe, exercise.

Based on 6 intervention arms, IF plus Exe decreased serum leptin significantly more than Exe only [SMD = −0.47 (95% CI −0.91 to −0.03), *p* = 0.03] (Figure 3). Among the included studies, there was no notable heterogeneity (*I*^2^ = 46.71%, *p* = 0.09). Examination of funnel plots and the results of the Egger’s test (*p* = 0.7) demonstrated the lack of publication bias. The sensitivity analysis was conducted by excluding individual studies. By excluding the (5) study during sensitivity analysis, the effect size and significance of the results were changed (SMD = −0.4, *p* = 0.1), but the overall findings remained consistent. Furthermore, when the (28) study was excluded, there were alterations in the effect size and significance (SMD = −0.5, *p* = 0.07), but the overall findings remained consistent.

##### 2.4.2.2 Adiponectin

**FIGURE 4:**
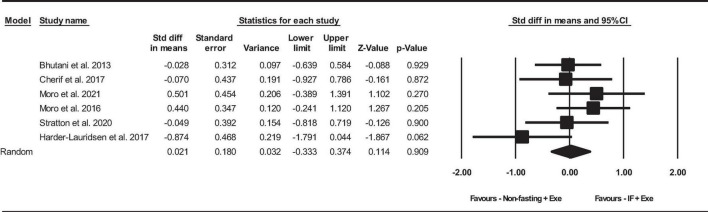
Forest plot of the effects of intermittent fasting plus exercise vs. exercise only on serum adiponectin. Data are reported as SMD (95% confidence limits). SMD, standardized mean difference; IF, intermittent fasting; Exe, exercise.

Based on 6 intervention arms, the combination of IF and Exe did not increase serum adiponectin significantly more than Exe only [SMD = 0.02, (95% CI −0.33 to 0.37), *p* = 0.9], as shown in Figure 4. Among the included studies, there was no notable heterogeneity (*I*^2^ = 20.94%, *p* = 0.2). The examination of funnel plots and the results of the Egger’s test (*p* = 0.5) did not identify any evidence of publication bias. Through the sensitivity analysis involving the exclusion of particular studies, it was observed that there were no modifications in the effect size, the significance of the findings, or the direction of the results.

### 2.5 Quality assessment

The PEDro tool was used to assess the methodological quality of each individual study, and scores varied between 4 and 7 out of a possible maximum of 9 points. One study had a score of 7 (18), three studies had scores of 6 (5, 25, 28, 41), one study had scores of 4 (1). Most of the PEDro scores were lowered due to two items (concealed allocation, and intention-to-treat analysis). The details of the quality analysis are shown in Supplementary Table 2.

## 3 Discussion

IF and Exe are well-established as non-pharmacological approaches to modulating different hormones and cytokines related to obesity (19, 42, 43). Our meta-analysis provides a better understanding of the effects of IF associated with Exe, and Exe alone, on serum concentrations of leptin and adiponectin. Overall, IF combined with Exe elicited a greater reduction in leptin levels with a small-to-moderate effect size, but did not change adiponectin. The changes in body weight were somewhat uncertain due to high heterogeneity and the small number of studies included. With all of the studies included, the benefit of the combination of IF and Exe for weight loss, as compared with Exe only, was bordering on significant, with a small difference in weight loss. Upon additional sensitivity analyses, the weight loss differences became statistically significant; however, these results should be considered with caution.

The decrease in leptin concentrations induced by Exe alone or combined with IF can occur both acutely and chronically due to the better sensitivity of the body to this adipokine (44). Regarding chronic effects, weight loss is known to promote improvements in the sensitivity of leptin and insulin, which explains the decrease in leptin after interventions with IF + Exe or isolated Exe (45, 46). The use of IF, Exe, and their combination as important interventions for reducing hypothalamic inflammation and enhancing leptin sensitivity. So that recent studies have suggested that the combination of both interventions as a more effective therapy for metabolic disorders (47). Although the mechanisms involved in the regulation of leptin through Exe are not yet well established, available data suggest that this could occur through the activation of different pathways and factors, such as PI3K/AKT, MAPK, mTOR, and PPARγ pathways (32). Furthermore, these reductions in leptin concentrations may be related to the secretion of cytokines, such as interleukin-6 (IL-6), since it is secreted by muscle tissue in response to intense Exe, tissue that also secretes leptin (43, 48). However, a possible acute influence of IL-6 on leptin has not been well established (48).

The levels of leptin are more responsive to a negative energy balance, meaning that leptin secretion is likely to decrease more in low-calorie diets compared to high-calorie diets. Additionally, it appears that leptin levels quickly return to baseline after the restoration of energy balance (49–51). Similarly, leptin levels decrease with high-protein diets, independent of increases in ghrelin levels (52). This indicates that despite the stable characteristics of leptin, baseline leptin levels may respond to short-term and long-term changes in dietary intake (8). Sustained elevations in circulating leptin levels have long-term implications for weight regulation, food reward systems, stress responses, and the potential development of neuropsychiatric behavioral disorders (8).

Contrary to our expectations, the combination of Exe and IF did not promote greater increases in adiponectin than Exe alone. Our hypothesis was based on evidence that both Exe and IF can reduce systemic inflammation and fat mass while ameliorating lipid and glucose metabolism, partially due to AMPK-related pathways, which favors an increase in adiponectin levels (32, 53–55). Regarding Exe *per se*, there are chronic and acute/transient increases in adiponectin via accumulation of lactate (acidosis), increased adrenaline secretion, and decreased glycogen storage (32, 45). Adiponectin responses to Exe occur with intense Exe (i.e., at or above the anaerobic threshold) of short durations (< 1 h) (45). Regarding chronic effects, improvements in body composition (increased muscle mass and decreased fat mass) via Exe can be considered the cornerstone of increasing adiponectin levels (32, 45).

In general, the combination of Exe and IF did not promote greater increases in adiponectin compared to Exe alone, such that individual results indicated a very small effect size, and lacked uniformity, likely due to the heterogeneity of the Exe protocols used. Among the six studies evaluated in this regard, two reported increases for IF + Exe using high-intensity resistance Exe (75–90% 1RM) (25, 28), and one reported increases following isolated Exe, using aerobic training (1). The two studies that demonstrated an advantage for IF + Exe used high-intensity resistance Exe (75–90% 1RM) (25, 28). Regarding the other three studies that did not demonstrate differences, one used resistance Exe (18), while the other two studies used aerobic Exe, one of which used a continuous protocol (5) and the other used repeated sprint training (RST) (41).

Beyond leptin and adiponectin levels, we also assessed body weight changes and showed that IF + Exe demonstrated greater reductions as compared with Exe alone. However, the heterogeneity in body weight changes was high, and these results should be interpreted with caution. This finding is in line with the recent meta-analysis of Khalafi et al. (56), which demonstrated that combining nutritional strategies with Exe was more efficient for weight loss than when either was applied alone (56). The heterogeneity of protocols in the included studies also occurs in the outcomes found. Bhutani et al. (5) used a continuous aerobic protocol (5), showing a tendency toward an advantage for IF + Exe with respect to weight loss, and an advantage for IF + Exe in relation to the reduction in leptin. The studies of Moro et al. (28) used high-intensity resistance Exe (25, 28), showing an advantage for IF + Exe on weight loss and reductions in leptin. Harder-Lauridsen et al. (1) showed an advantage for IF + Exe in relation to weight loss, and a trend toward an advantage over adiponectin secretion for this intervention (1). The protocols employed by Cherif et al. (41) and Stratton et al. (18) did not demonstrate an advantage in any of the evaluated parameters, whether for IF + Exe or isolated Exe (18, 41).

The present study was novel, comparing the effects of Exe alone or in combination with IF regimens, not only on weight loss, but also on leptin and adiponectin levels, important for regulating metabolism and promoting health. In contrast, our study has some limitations. First, most studies included only men; only the study by Bhutani et al. (5) included both men and women (5). Furthermore, the interventions were brief, ranging from 3 days to 12 weeks, except the study by Moro et al. (25), which lasted 52 weeks (25). Most studies evaluated only healthy people, with only Bhutani et al. (5) evaluating men and women with obesity (5). Furthermore, the included studies demonstrated that both aerobic and resistance Exe can be efficient when combined with IF for weight loss. However, one must note the preservation of muscle mass is an important variable when designing a training program, as it has a direct relationship with health in general, in addition to maintaining efficient metabolic processes for weight loss (57, 58). Finally, the quality of the diet could not be determined in this meta-analysis and may have been an important factor for determining anthropometric and biochemical changes (54, 59).

One strength of the current systematic review and meta-analysis is a low publication bias. In addition, we diminished potential bias in the review process by performing a comprehensive search of the literature and also by conducting and reporting the systematic review and meta-analysis according to the PRISMA guidelines. In addition, no significant heterogeneity was observed for leptin and adiponectin.

The current systematic review and meta-analysis had several limitations that should be considered when interpreting the results. There was only a small number of studies that met the inclusion criteria with adipokine outcomes available. Therefore, we were not able to compare the effects of specific IF or Exe regimens. Also, it was not possible to perform subgroup analyses based on the age, body weight, BMI of the subjects, or duration of the IF and Exe interventions. Finally, because of the available eligible studies, the present study was limited to young adults, and with the increasing number of new primary research studies, future meta-analyses that include middle-aged and older adults are needed.

## 4 Conclusion

The current meta-analysis indicated that IF combined with Exe reduced leptin, but did not change adiponectin levels, when compared to Exe alone. Perhaps these reductions in leptin levels may have been associated with weight loss; however, due to the small number of included studies and the high heterogeneity in the weight loss outcomes, this result is uncertain.

## Data availability statement

The raw data supporting the conclusions of this article will be made available by the authors, without undue reservation.

## Author contributions

FK: Conceptualization, Data curation, Formal analysis, Investigation, Methodology, Software, Supervision, Writing – original draft, Writing – review & editing. NB: Data curation, Methodology, Writing – original draft. HC: Writing – review & editing. HS: Methodology, Writing – review & editing. SR: Writing – review & editing.
