# Supplementary material for: Effects of intermittent fasting combined with exercise on serum leptin and adiponectin in adults with or without obesity: a systematic review and meta-analysis of randomized clinical trials
Source: Front Nutr. 2024 Jun 12;11:1362731. doi: 10.3389/fnut.2024.1362731 (PMC11199738; doi:10.3389/fnut.2024.1362731)
Supplement: Supplementary file 1 [file Table_1.docx]

| Supplementary Table 1. Search strategy | | | |
| --- | --- | --- | --- |
| Databases | Search strategy | Limits | Results |
| PubMed | (("Intermittent energy restriction" or "intermittent caloric restriction" or "intermittent fasting" or "fasting" or "intermittent energy" or "intermittent calorie" or "intermittent diet" or "time-restricted feeding" or "Time Restricted eating" or "alternate-day fasting" or "alternate day fasting" or "alternate day diet" or "Ramadan fasting") AND ("Exercise" or "training" or "physical activity" or "exercise training" or "sport" or "strength training" or "strength exercise" or "weight training" or "resistance training" or "progressive training" or "progressive resistance" or "weightlifting" or "aerobic exercise" or "aerobic training" or "endurance exercise" or "endurance training" or "cardio training" or "physical endurance" or "physical exertion")) AND ("adipokine" or "adipocytokine" or "leptin" or "adiponectin") | Human, English | 670 |
| Scopus | (("Intermittent energy restriction" or "intermittent caloric restriction" or "intermittent fasting" or "fasting" or "intermittent energy" or "intermittent calorie" or "intermittent diet" or "time restricted feeding" or "Time Restricted eating" or "alternate-day fasting" or "alternate day fasting" or "alternate day diet" or "Ramadan fasting") AND ("Exercise" or "training" or "physical activity" or "exercise training" or "sport" or "strength training" or "strength exercise" or "weight training" or "resistance training" or "progressive training" or "progressive resistance" or "weightlifting" or "aerobic exercise" or "aerobic training" or "endurance exercise" or "endurance training" or "cardio training" or "physical endurance" or "physical exertion")) AND ("adipokine" or "adipocytokine" or "leptin" or "adiponectin") | Article, English | 978 |
| Web of Science | ((TS=("Intermittent energy restriction" or "intermittent caloric restriction" or "intermittent fasting" or "fasting" or "intermittent energy" or "intermittent calorie" or "intermittent diet" or "time restricted feeding" or "Time Restricted eating" or "alternate-day fasting" or "alternate day fasting" or "alternate day diet" or "Ramadan fasting")) AND TS=("Exercise" or "training" or "physical activity" or "exercise training" or "sport" or "strength training" or "strength exercise" or "weight training" or "resistance training" or "progressive training" or "progressive resistance" or "weightlifting" or "aerobic exercise" or "aerobic training" or "endurance exercise" or "endurance training" or "cardio training" or "physical endurance" or "physical exertion")) AND TS=("adipokine" or "adipocytokine" or "leptin" or "adiponectin") | Article, English | 884 |
