# Supplementary material for: Effects of intermittent fasting combined with exercise on serum leptin and adiponectin in adults with or without obesity: a systematic review and meta-analysis of randomized clinical trials
Source: Front Nutr. 2024 Jun 12;11:1362731. doi: 10.3389/fnut.2024.1362731 (PMC11199738; doi:10.3389/fnut.2024.1362731)
Supplement: Supplementary file 2 [file Table_2.docx]

Supplementary Table 2: Risk of bias assessment (PEDro scale)

| Authors and Year of Publication | Criteria 1 | Criteria 2 | Criteria 3 | Criteria 4 | Criteria 5 | Criteria 6 | Criteria 7 | Criteria 8 | Criteria 9 | Total |
| --- | --- | --- | --- | --- | --- | --- | --- | --- | --- | --- |
| Bhutani et al., 2013 (Bhutani et al., 2013b) | 🗸 | 🗸 | × | × | × | 🗸 | 🗸 | 🗸 | 🗸 | 6 |
| Cherif 2017 (Cherif et al., 2017) | 🗸 | 🗸 | × | × | 🗸 | 🗸 | × | 🗸 | 🗸 | 6 |
| Moro et al., 2021 (Moro et al., 2021a) | 🗸 | 🗸 | × | 🗸 | 🗸 | × | × | 🗸 | 🗸 | 6 |
| Moro et al., 2016 (Moro et al., 2016a) | 🗸 | 🗸 | × | 🗸 | 🗸 | × | × | 🗸 | 🗸 | 6 |
| Stratton et al., 2020 (Stratton et al., 2020a) | 🗸 | 🗸 | × | 🗸 | × | 🗸 | 🗸 | 🗸 | 🗸 | 7 |
| [Harder-Lauridsen](https://pubmed.ncbi.nlm.nih.gov/?term=Harder-Lauridsen+NM&cauthor_id=28359370) et al., 2017 (Harder-Lauridsen et al., 2017a) | 🗸 | × | × | ? | × | 🗸 | × | 🗸 | 🗸 | 4 |
